# Relevance of breast MRI in determining the size and focality of invasive breast cancer treated by mastectomy: a prospective study

**DOI:** 10.1186/s12957-017-1197-1

**Published:** 2017-07-14

**Authors:** Anne-julie Carin, Sébastien Molière, Victor Gabriele, Massimo Lodi, Nicolas Thiébaut, Karl Neuberger, Carole Mathelin

**Affiliations:** 1 CARIN Anne-Julie Centre hospitalier de Haguenau, 64 avenue du Professeur Leriche, 67500 Haguenau, France; 2 MOLIERE Sébastien MD Unité d’imagerie mammaire CHRU Hôpitaux universitaires de Strasbourg, Avenue Molière, 67200 Strasbourg, France; 3 GABRIELE Victor Unité de sénologie CHRU Hôpitaux universitaires de Strasbourg, Avenue Molière, 67200 Strasbourg, France; 4 LODI Massimo Unité de sénologie CHRU Hôpitaux universitaires de Strasbourg, Avenue Molière, 67200 Strasbourg, France; 5 THIEBAUT Nicolas, statisticien - QUANTMETRY, 128 rue du Faubourg St-Honoré, 75008 Paris, France; 6 NEUBERGER Karl, QUANTMETRY, 128 rue du Faubourg St-Honoré, 75008 Paris, France; 7 MATHELIN Carole MD PhD Unité de sénologie CHRU Hôpitaux universitaires de Strasbourg, Avenue Molière, 67200 Strasbourg, France; 8 Department of Functional Genomics and Cancer, Institut de Génétique et de Biologie Moléculaire et Cellulaire (IGBMC), CNRS UMR 7104, INSERM U964, Université de Strasbourg, Illkirch, France

**Keywords:** Invasive breast cancer, Breast MRI, Multiple foci, Tumour size, Histology

## Abstract

**Background:**

The aim of this study was the evaluation of breast MRI in determining the size and focality of invasive non-metastatic breast cancers.

**Methods:**

The prospective, single-centre study conducted in 2015 compared preoperative MRI with histological analysis of mastectomy.

**Results:**

One hundred one mastectomies from 98 patients were extensively analysed. The rates of false-positive and false-negative MRI were 2 and 4% respectively. The sensitivity of breast MRI was 84.7% for the detection of all invasive foci, 69% for single foci and 65.7% for multiple foci. In the evaluation of tumour size, the *Spearman rank correlation coefficient r* between the sizes obtained by MRI and histology was 0.62. The MRI-based prediction of a complete response to neoadjuvant chemotherapy was 75%.

**Discussion:**

MRI exhibits high sensitivity in the detection of invasive breast cancers. False positives were linked to the inflammatory nature of the tumour bed. False negatives were associated with small or low-grade tumours and their retro-areolar location. The size of T1 tumours was overestimated by an average of 7%, but MRI was the most efficient procedure. The sensitivity of MRI for the diagnosis of unifocal tumours was higher than that for multifocal sites. Our study confirmed the positive contribution of preoperative MRI for invasive lobular carcinomas and complete response predictions after neoadjuvant chemotherapy.

## Background

Preoperative assessment of the size and focal nature of invasive breast cancer is essential for the establishment of the global therapeutic strategy and optimized choice of surgery. The goal of the surgeon is twofold: first, the choice between performing mastectomy or more conservative treatments providing a satisfactory cosmetic outcome while minimizing possible repeat surgery for residual cancerous tissue and, second, to choose the optimal timing of this surgery, which can be initial or following neoadjuvant chemotherapy (NAC).

Clinical examination, mammography and breast ultrasound are currently the benchmark exams in the estimation of tumour size [1] and in multifocal tumour (MFT) diagnosis. MRI is usually recommended in cases of discrepancy between clinical, mammography and ultrasound before certain specific therapeutic approaches are used (oncoplastic surgery, NAC) in young women or with a high family risk of breast cancer; in the event of MFT, it can also be provided for evaluation of the contralateral breast [2]. However, the routine use of MRI in breast cancer is not recommended [3]. Some studies have shown that it can lead to higher rates of immediate mastectomy in patients initially planned for conserving surgery [4]. Other teams have shown that MRI did not always result in reduced rates of repeat surgery [3, 5]. The benefit of using breast MRI in the evaluation of tumour size, as well as in the diagnosis of MFT, compared to histological data is currently the subject of controversy [6–11].

The aim of our prospective study was to compare data obtained from preoperative breast MRI with those of cytopathological analyses of breast specimen as regards the focal nature and size of the tumour foci, for a continuous series of patients treated for invasive non-metastatic breast cancer by total mastectomy in 2015.

## Methods

We conducted a prospective, single-centre study in the Breast Pathology Unit of the University Hospital of Strasbourg (UHS), including all patients who underwent mastectomy for primary invasive breast cancer with prior examination by MRI, during the year 2015.

### Inclusion criteria

All patients selected for the study underwent unilateral or bilateral mastectomy for invasive non-metastatic breast cancer, coupled with breast MRI for preoperative assessment of local invasion. Patients who underwent NAC involving breast MRI re-evaluation before surgery were also included. MRI was performed either in the imaging department of the UHS or in an external radiology practice. Histological examination of surgical specimens all took place in the UHS Department of Pathology. The radiological and histological analyses focused on the operated breast.

### Exclusion criteria

The absence of preoperative breast MRI, mastectomies for non-invasive cancer, prophylactic mastectomies without invasive foci found by cytopathology, repeat mastectomies for unhealthy margins after conservative surgery, and metastases at baseline were the exclusion criteria.

Written consent of each patient was obtained (registration number 1187586 file of the Data Protection Commission).

For each patient included, the following medical data were recorded: age, breast surgery history, gene mutation predisposing to breast cancer, invasive cancer type, tumour grade and immunohistochemical characteristics (oestrogen receptor, progesterone receptor, expression of the human epidermal receptor 2, tumour Ki67 proliferation index), number and size of the invasive foci as seen by both histology and MRI, and the presence of ductal carcinoma in situ (DCIS) in proximity to or remote from the invasive component. In the presence of MFT, we recorded the pathological characteristics of the largest focus unless a smaller focus presented a worse aspect. If NAC was performed, the radiological data corresponded to breast MRI after chemotherapy, and only these data were compared with histologic findings.

### Breast MRI procedure

All patients in the study underwent breast MRI in the month before mastectomy, with one patient operated due to hereditary predisposition to breast cancer for which MRI took place 5 months before surgery. Initially, scanning sequences without injection were performed (T1 and T2), followed by thin slices (<3 mm) gadolinium-enhanced sequences. Image analysis involved the use of subtraction techniques, multiplanar reconstruction and dynamic enhancement curves. The number of breast biopsies after MRI-based recommendations was recorded, as well as their side (ipsi- or contralateral) and the result of pathological analysis.

### Histological analysis

All mastectomy samples were fixed in 4% formalin solution for 12 to 24 h, then dried under vacuum in an automated embedding device. These samples were subsequently embedded in paraffin wax, cut by microtome, stained and mounted on microscope slides.

We compared the preoperative breast MRI data with pathological data obtained from large-format histopathology slides in the evaluation of the uni- or multifocal character of the tumour and the size of the principal foci. To allow for tissue distortion due to processing, radio-histological correlation was calculated at 10 and 20% of the size given by cytopathology. Measurement of the greater axis from tumour histological analysis was used to calculate upper and lower boundaries corresponding to tumour diameter, plus and minus 10 or 20%. When the size given by MRI fell between these two limits, concordance was affirmed. Benign histological lesions, such as fibroadenoma, papilloma, fibrocystic or fibro-inflammatory changes, were also noted.

### Statistical analysis

The accuracy of measuring size of tumour foci by breast MRI was evaluated in different sub-groups. The differences between these sub-groups were analysed by the *Fisher exact test* with regard to the number of foci, as well as the paired samples *Student t test* with regard to tumour size. Correlation between MRI and histological size was performed using the *Spearman rank correlation coefficient*.

## Results

### Population studied

During 2015, 958 breast cancers were treated in our surgical department, for which 364 total mastectomies were performed. In our study, 98 patients met the inclusion criteria, i.e. 101 mastectomies. In this cohort, the mean age of patients was 58 years (25–83).

We included 34 patients (33.7%) who underwent NAC, and 64 patients (corresponding to 67 mastectomies (62.4%)) treated from the outset by surgery. Two patients (1.9%) were operated in the context of *BRCA2* gene mutations. Three patients (3%) were operated for a double mastectomy for synchronous bilateral breast cancer.

### Histological and immunohistochemical characteristics of tumours

Cytopathological analysis of specimens identified 176 infiltrating cancer sites, while preoperative breast MRI detected 148, a sensitivity of 84.1%. The primary tumour was invasive ductal carcinoma (IDC) in 80 cases (79.2%), invasive lobular carcinoma (ILC) in 17 cases (16.8%), mixed carcinoma combining IDC and ILC in 2 cases (2%), and papillary carcinoma and a micropapillary carcinoma in the last two cases (2%). A DCIS component was found in 47 cases (46.5% of surgical specimens).

The characteristics of the main tumours are described in Table 1.

**Table 1:** Pathological and immunohistochemical characteristics of tumours. For 8 patients, due to complete response to NAC, these characteristics refers to the pre-NAC core needle biopsy

	IDC *n* = 80	ILC *n* = 17	MC *n* = 2	IPC *n* = 1	MPC *n* = 1
SBR grade					
1	9	5	0	0	0
2	44	9	2	1	0
3	27	3	0	0	1
ER/PR					
Positives	61	17	2	1	0
Negatives	19	0	0	0	1
HER2					
0	51	13	2	0	0
1+	9	2	0	0	0
2+ NA	8	1	0	1	0
2+ A	1	1	0	0	0
3+	11	0	0	0	1
Ki67					
<10%	9	6	1	0	0
10–30%	44	11	0	1	1
>30%	26	0	1	0	0
NR	1	0	0	0	0

*ER* oestrogen-receptor, *PR* progesterone-receptor, *IDC* invasive ductal carcinoma, *ILC* invasive lobular carcinoma, *MC* mixed carcinoma (combining IDC and ILC), *IPC* invasive papillary carcinoma, *MPC* invasive micropapillary carcinoma

### Concordance between MRI and histology data

#### Tumour size

**Fig. 1:**
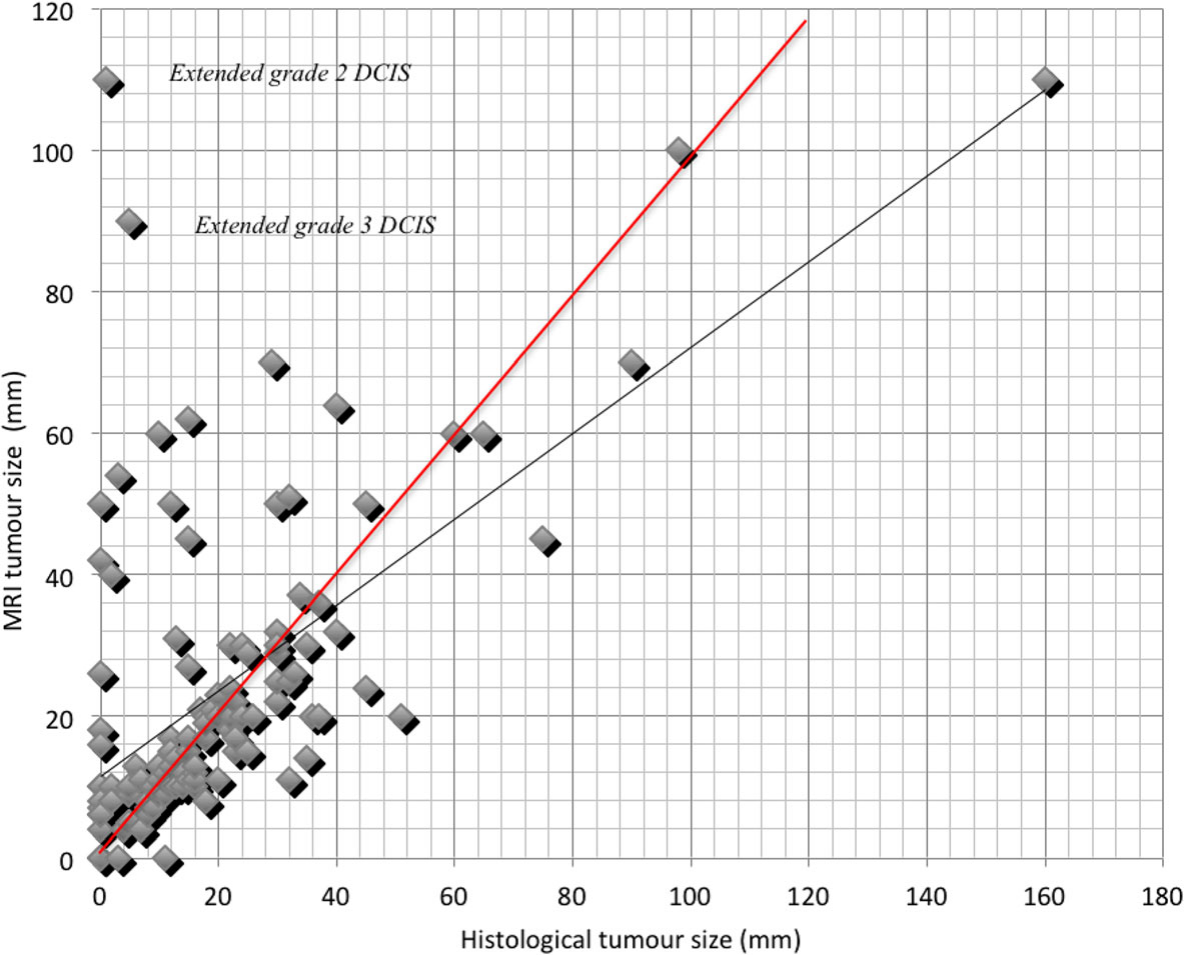
*Scatter plot* shows the relationships between tumour size determined on microscopic and on MRI examination in millimetres. The *red line* underlines the perfect match (ideal relationship) between MRI size and pathological size (reference size). The *grey line* represents our cohort

The distribution of tumour sizes identified by MRI compared with those measured by histology (linear correlation) is described in Fig. 1. The concordance was 19.4% with a threshold of 10% and 31.8% with a threshold of 20%. With this second threshold, lesion size was underestimated by MRI in 24/117 cases (20.5%) and overestimated in 28/117 cases (23.9%). A sub-group analysis was performed to identify possible factors influencing the correlation between MRI and cytopathology. In 56.5% of cases where MRI overestimated tumour size of the main focus (13/23), there was associated DCIS, and high-grade DCIS was associated with an overestimation of invasive tumour size by MRI (*p* < 0.005). In analysis of the grade of the main tumour present in each mastectomy sample, 20% concordance was found in 31/70 (44.2%) for grade 1 and 2 tumours, and only 12/31 (38.7%) for grade 3 tumours (*p* = 0.82). The concordance size of 20% was higher for ILC (58.8%) than for IDC (39.0%), without this difference reaching statistical significance.

Evaluation of tumour size was significantly different among T1 tumours (≤2 cm) and larger tumours (T2, T3 and T4 tumours >2 cm): MRI overestimated the first group by an average of 7.1% and underestimated the second group on average 14.5% (*p* = 0.0001). Overall, the size of tumours <2 cm was overestimated by MRI in 31.8% of cases and underestimated in 22.7% of cases. For tumours >2 cm, the rate of over- and underestimation were 21.4 and 57.1% respectively. We found a *Spearman rank correlation coefficient r* = 0.62 (*p* < 0.0001) for the entire cohort.

#### Focality

Cytopathological analysis of 101 surgical specimens identified between 0 and 12 invasive cancer foci. A single-invasive focus was found in 58 cases (57.4%) and MFT in 35 cases (34.7%). A pathological complete response to NAC was seen in 8 cases (23.5%). In the case of MFT, histological analysis of the primary tumour led to diagnosis of IDC in 23 cases (28.5% of all IDC) and ILC in 10 cases (58.8% of all ILC).

### Validity of breast MRI (false-positives to false-negatives)

MRI wrongly suspected invasive cancer in two tumour-free surgical specimens (2% of the series). In both cases, patients had received NAC resulting in complete recovery, but in one case, MRI revealed an extended low-intensity mass enhancement of 42 mm and in the other case a combined low-intensity mass of 6 mm and a non-mass-like enhancement of 26 mm.

Conversely, MRI wrongly failed to detect suggestive images of invasive cancer in four cases (4%). In the first case, after NAC, the residual infiltrating focus measured 16 × 12 mm. In the second case, the patient was treated in the context of a *BRCA2* mutation and histology demonstrated a high-grade IDC 11 mm focus. The third case involved a bifocal cancer with two low-grade 5 and 4 mm foci. Finally, in the last case, there was a single-centre retro-areolar grade 1 ILC of 55 × 50 × 35 mm.

#### Unifocality

MRI indicated unifocality in 52 cases (42 IDC, 9 ILC, 1 micropapillary carcinoma) confirmed by cytopathology in 40 cases (76.9%), i.e. an MRI efficiency of 69% in the diagnosis of unifocality (40/58). MRI underestimated focality (additional invasive foci seen in samples examined by histology) in 11/52 cases (21.1%): 7 and 4 for IDC or ILC respectively. It overestimated focality (no focus seen by histology) in 1/52 cases (1.9%): 1 IDC.

#### Multifocality

Multifocality was suspected in 39 cases with histological confirmation of several invasive foci in 23 cases (59%). The sensitivity of MRI in the diagnosis of MFT was therefore 65.7% (23/35). In 16 cases where MRI suggested excessive multifocality, cytopathological analysis revealed either no tumour or a single focus (*n* = 66), a false-positive rate of 24.2%.

Fifteen breast biopsies in 14 patients (14%) were done after MRI-based recommendations, 9 in the breast of the index tumour, 5 in the contralateral breast and 1 in screened high-risk breasts. The result of pathological analysis was malignant in 10 biopsies for 9 patients (9%) and benign in 5 biopsies for 5 patients (5%). Malignant findings included ductal invasive carcinoma (*n* = 8), lobular invasive carcinoma (*n* = 1) and DCIS (*n* = 1). Average size of MRI-detected additional malignant lesions was 7 mm (minimum 4 mm, maximum 12 mm). All of this lesions were distant of more than 1.5 cm from the index tumour. Benign findings included fibrosis (*n* = 1), fibrocystic changes (*n* = 3) and fibroadenoma (*n* = 1).

#### Number of foci

In 54/101 cases (53.5%), MRI was concordant with histological analysis as regards the number of invasive foci: 69% unifocal tumours (40/58) and 22.9% multifocal tumours (8/35). MRI overestimated and underestimated the number of invasive lesions in 18.8% (19/101) and 27.7% (28/101) of cases, respectively. The presence of associated DCIS (observed in 7/19 cases) was not found to be a factor explaining this overestimation (*p* = 0.80). Benign lesions, such glandular fibrocystic changes or marked fibro-inflammatory changes after neoadjuvant therapy, were more frequently seen at pathological examination associated with MRI overestimation of the number of malignant foci (17/19, *p* = 0.02). The MRI-pathology concordance was 62.1% for high-grade carcinomas and 48.6% for other grades (*p* = 0.27). Regarding the histological type, histo-radiological comparison was correct in 8/17 cases (47.1%) for ILC and 55% for IDC (44/80 cases) (*p* = 0.59).

In the sub-group of patients treated with NAC (*n* = 34), MRI was consistent with cytopathology in 21 cases (61.8%), including prediction of complete recovery in 6/8 cases (75%). In a single case (2.9%), MRI erroneously failed to detect a residual invasive focus of 16 mm. Tables 2 and 3 summarize the histological and radiological results in terms of number of foci.

**Table 2:**
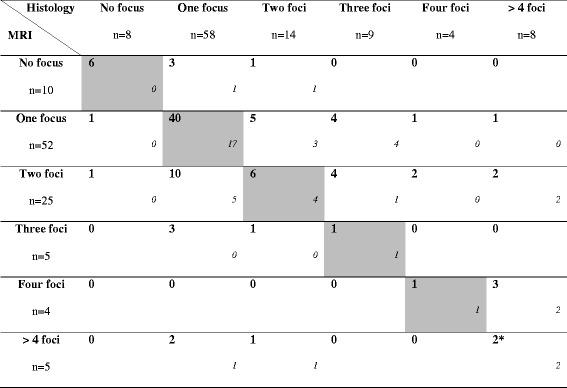
Histological and breast MRI focality diagnosis (number of mastectomy specimens with the presence of associated DCIS to invasive component is specified in italics). Concordant cases are greyed out

^*^MRI was discordant with histological analysis as regards the number of invasive foci (underestimated by MRI)

**Table 3:**
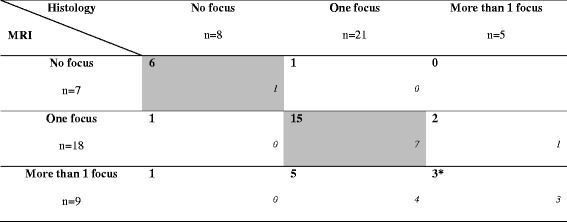
Histological and Breast MRI focality diagnosis after NAC (number of mastectomy specimens with the presence of associated DCIS to invasive component is specified in italics). Concordant cases are greyed out

^*^MRI was discordant with histological analysis as regards the number of invasive foci (underestimated by MRI)

## Discussion

In our prospective study, the overall *sensitivity* of MRI in detecting invasive tumour foci is high (84%). Our results are consistent with published literature which also report high sensitivity of MRI, varying between 71 and 100% [12]. The originality of our study was the inclusion of patients treated exclusively by total mastectomy and for which large-section histopathology data were available, guaranteeing complete coverage in the detection of multiple tumour foci, while other authors also included patients treated conservatively (for which the entire mammary gland was not analysed).

The *false-positive rate* of preoperative MRI was very low (2%) in our study. The further analysis of two cases in which MRI showed the persistence of a low-intensity enhancement without histological confirmation of a residual tumour was linked to the inflammatory nature of the tumour bed. Our results support the use of preoperative histological analysis to verify tumour presence suspected by MRI [13, 14]; information given to patients should stress the rate of false-positive MRI, seen to be substantially lower in our series compared to the literature where rates vary between 9.9 and 26% [13, 15].

The *false-negative rate* of preoperative MRI was also very low, 4% in our study. Regarding these four patients, in one case, we were unable to explain the failure to detect a 16 × 12 mm focus after NAC. However, in three cases, tentative explanations exist for the absence of tumour in MRI images: for the patient with a *BRCA2* mutation, the extended 5 months period between imaging and surgery may explain the lack of detection. Another patient had small tumours (4 and 5 mm), and it is now accepted that the diagnostic performance of MRI drops below a 5-mm threshold [16]. In the last case (grade 1 retro-areolar carcinoma of 55 mm), we speculate that the combination of the retro-areolar anatomy complicating analysis (physiological enhancement) and low enhancement of low-grade tumours led to failed detection.

In the case of *NAC*, MRI has proven to be very effective, especially in predicting complete recovery (75% agreement). Its contribution in assessing the response to NAC holds promise for many teams [17–19] with predictions comparable to our study, around 72–74% [18, 20]. Nevertheless, Vriens et al. [21] recently warned about the relatively low negative predictive value of this exam (26%) for hormone-sensitive tumours. In this cited study, subsequent to NAC 74% of patients in which MRI did not reveal signs of malignancy actually possessed residual tumour. In our series, we found only one case (2.9%) where breast MRI did not detect the residual infiltrating focus, and it was hormone-sensitive.

Regarding *tumour sizes*, in our study, MRI on average overestimated tumours <2 cm by 7% and underestimated tumours >2 cm by 14.5%. For some physicians, breast MRI is the most accurate examination [1, 13, 22, 23]; others prefer conventional imaging which they contend allows to most accurately approximate tumour size [6, 24]. Table 4 summarizes the performance of different imaging studies in estimating tumour size. The tendency to overestimate lesion size by breast MRI was found by several authors [6, 10, 25, 26] and in more than 50% of the cohort of 682 patients of Lai et al. [26]. This is partly related to the method of measurement of tumour size and the concordance thresholds established prior to examination. Indeed, the types of tumour tissue available to the pathologist may influence the determination of tumour size. When measurement is conducted on a fresh specimen, tumour size is always greater than that determined from fixed ones, with tissue shrinkage related to fixation and processing being estimated at 10% of the total volume for mainly fibrous tissue, 25% for primarily adipose tissues and around 20% for “intermediate” histological specimens [27]. Because of this difference, we chose a 20% concordance value between MRI and histology, rather than an absolute figure. Unlike other teams, we found it more informative to establish a percentage match threshold of histological tumour size and not a fixed difference regardless of cancer size (roughly 5 mm [7, 10] or 10 mm [8] of the size determined by histology). Indeed, this calculation allows us to have very similar real measurements for small tumours (e.g. for a 4-mm tumour, the margin of 20% corresponds to an estimated size of 3.2 to 4.8 mm). This accuracy is critical for the clinician, knowing that pT1a tumours require special attention, usually without adjuvant therapy.

**Table 4:** Tumour size estimation in the literature: radiological and pathological correlation

Author year (reference)	Histological sub-type	Patients (*n*)	Mammography	Ultrasounds	MRI
Mann 2008 [33]	ILC	67	*r* = 0.27		*r* = 0.85
Wasif 2009 [1]	IDC, ILC, DCIS, others	61	*r* = 0.26	*r* = 0.57	*r* = 0.80
Ramirez 2012 [24]	Invasive carcinoma	161	*r* = 0.76	*r* = 0.67	*r* = 0.75
Lafaye-Carré 2014 [13]	IDC, ILC, CIS, IPC	89	ND	*r* = 0.45	*r* = 0.68
Rudat 2015 [22]	Invasive carcinoma	64	ND	*r* = 0.66	*r* = 0.77
Leddy 2016 [6]	DCIS, IDC, ILC	57	CCC = 0.58	CCC = 0.71	CCC = 0.50
UH Strasbourg 2016	Invasive carcinoma	98	ND	ND	*r* = 0.62

*IDC* invasive ductal carcinoma, *ILC* invasive lobular carcinoma, *DCIS* ductal carcinoma in situ, *CIS* carcinoma in situ, *IPC* invasive papillary carcinoma, *r* correlation coefficient, *CCC* Lin’s concordance correlation coefficient, *ND* not determined

Other authors [8, 10] have also reported equivalent proportions of over- and underestimation of tumour size. While in our study, the size was underestimated in 20.5% and overestimated in 23.9% of cases, Haraldsdottir et al. [8] and Grimsby et al. [10] underestimated size in 4.6 and 15% of cases and overestimated in 7.5 and 33% of cases, respectively. The presence of a single DCIS [7, 28] or associated with an invasive component was cited as a possible cause of overestimation of tumour size [10]; in our study, this was only found for high-grade DCIS, which is known to enhance on MRI, even in the lack of neoangiogenesis [29]. As regards tumour grade, some authors [30, 31] have scored significantly higher concordance for high-grade tumours, due to the usually rounded shape of these cancers facilitating measurement. In contrast, others [7] showed that high-grade carcinomas were more often overestimated in size by MRI. This was not observed in our study involving 31 high-grade foci. For some authors, histological type is associated with a significant overestimation of tumour size by MRI, as seen for IDC [6, 25] and ILC [25]. However, these results are the subject of controversy [10, 32]. In our series, we noted more frequent size mismatches for IDC.

In *the diagnosis of MFT*, breast MRI shows high sensitivity, reported as 83% by Rudat et al. [22]; it successfully detected 9% of tumours, otherwise not seen by conventional imaging for Girardi et al. [9]. In our study, the sensitivity of breast MRI in the diagnosis of MFT was lower, close to 66%. This difference is possibly due to our histological analysis of mastectomy specimens, the only guarantee of a comprehensive analysis of the mammary gland, whereas other authors included samples obtained from both mastectomy and lumpectomy. Nevertheless, there were 10 biopsy-proved additional malignancies detected only by MRI in 9 patients (9%). These additional lesions were all located more than 1.5 cm from the index tumour, potentially leading to modification of surgical planification.

Less frequent, overestimation of multifocality by MRI, while pathological analysis revealed either no tumour or a single focus, is thought to be secondary to enhancement of benign tumours, post-chemotherapy fibro-inflammatory changes or glandular fibrocystic changes. In these cases, especially when the possibility of additional malignancy may change the surgical planification, further evaluation, including second-look mammography, ultrasound and ultimately core-needle biopsy, is mandatory.

## Conclusion

Besides confirming the excellent positive and negative predictive value of MRI for detection of invasive lesions, the correlation between 101 whole-breast large-section histopathology datasets and preoperative MRI in our study indicates that MRI allows accurate estimation of the tumour size and focality. MRI-recommended biopsies allowed detection of additional distant malignancies in 9% of the patients.

As previously reported, MRI is especially useful for evaluation of ILC and to appraise complete pathological response after NAC.

MRI interpretation may be cautious in the presence of enhancing high-grade DCIS, which may impair accurate evaluation of the invasive component. Benign lesions such as post-chemotherapy fibro-inflammatory changes or glandular fibrocystic changes may also alter the evaluation of focality by MRI. Comprehensive post-MRI imaging assessment, including second-look ultrasound and second-reading of mammograms, as well as core-needle biopsy, may drastically reduce the consequences of potential MRI false positive.

## Abbreviations

DCIS: Ductal carcinoma in situ
IDC: Invasive ductal carcinoma
ILC: Invasive lobular carcinoma
MFT: Multifocal tumour
MRI: Magnetic resonance imaging
NAC: Neoadjuvant chemotherapy
